# Interpopulational Variations of Odorant-Binding Protein Expression in the Black Cutworm Moth, *Agrotis ipsilon*

**DOI:** 10.3390/insects11110798

**Published:** 2020-11-13

**Authors:** Jean-François Picimbon

**Affiliations:** 1 Biotechnology Research Center, Shandong Academy of Agricultural Sciences, Jinan 250100, China; jfpicimbon@gmail.com; Tel.: +86-531-83175350; Fax: +86-531-78156; 2 School of Bioengineering, QILU University of Technology, Jinan 250353, China

**Keywords:** noctuid, antennal binding protein X, general odorant-binding protein, calreticulin, migration, host-plant selection, memorization

## Abstract

**Simple Summary:**

Odorant-binding proteins (OBPs) are small soluble transporter proteins that are believed to play a key role in insect olfaction. However, there is an emerging set of data that shows a role in insecticide resistance for similar families of binding proteins. The black cutworm *Agrotis ipsilon* is a migrant species of moth known to feed on multiple types of crops (polyphagous) worldwide. It is therefore likely that the olfactory system of this species can be modulated to adapt to different environments. We compared gene expression between American and European continental populations of the moth. We found continental-specific expression of antennal binding protein X (ABPX) and general odorant-binding protein 2 (GOBP2), suggesting a function of these proteins in migration, environment recognition, crop change and adaptation that are required for a polyphagous species such as *A. ipsilon*.

**Abstract:**

A long-range migrant species of moth (*Agrotis ipsilon*) has served as a model to compare the expression profiles of antennal proteins between different continental populations. Our results showed that the American and French populations of the black cutworm moth, *A. ipsilon*, expressed the same odorant-binding proteins (OBPs), but apparently in different levels. Electrophoretic analysis of antennal protein profiles and reverse transcription polymerase chain reaction using RNA as a template showed significant differences between the two populations in the expression of antennal binding protein-X (ABPX) and general odorant-binding protein-2 (GOBP2). However, the two *A. ipsilon* populations showed no differences in RNA levels coding for pheromone binding proteins (PBPs), suggesting that the expression of generalist OBPs is population-specific and could be affected by specific odor and/or chemical changes in external environmental conditions. To support the role of ABPX and GOBP2 with expression, the role of ABPX and GOBP2 is discussed in regard to odor detection, memorization and/or degradation of toxic chemical insecticides.

## 1. Introduction

Most animals, including insects, use odors in different behavioral contexts, e.g., to find a mating partner, food sources, a habitat, or oviposition sites. In moths, males are attracted by sex pheromones produced by conspecific females. Moth larvae orientate and feed preferentially on the diet on which they have been reared; food preferences and host plant recognition are dependent on olfactory cues [1,2,3,4,5]. Food (plant)-odor-detecting neurons, first classified as generalists, often respond to a wide spectrum of odors at high concentrations. In fact, it has been described that all insect chemoreceptors display a high specificity and sensitivity for the key-stimulus, including plant volatiles [6,7]. The “generalist odorant”-sensitive sensilla (or sensory hairs) associate specific neurons with specificity and sensitivity comparable to pheromone receptors, and in some cases, they pair receptor cell types specific to host semiochemicals and receptor cell types tuned to non-host semiochemicals [8,9,10].

Plant odor-sensitive and pheromone-sensitive cells show the same morphology within a sensillum, the micro-organ involved in odor detection in insects. Like in the moth pheromone-sensitive model of sensilla trichodea, the neuronal dendrites in the sensilla basiconica are bathed in the proteinaceous lymph that entirely fills the sensillar cavity [11]. Plant odor and pheromone molecules, largely hydrophobic in nature, must then cross this lymph before interacting with neurons. Small water-soluble odorant-binding proteins (OBPs), in extreme concentrations in the lymph, facilitate solubilization, transport and delivery of odorant molecules to the receptor neurons [12,13,14,15,16]. A great (more than two thousand) number of OBPs in Lepidoptera has been identified across different genera and species and deposited in NCBI database (https://www.ncbi.nlm.nih.gov). Moth OBPs are mainly classified into pheromone-binding proteins (PBPs) and generalist odorant-binding proteins (GOBPs) on the basis of selective expression in sensilla trichodea or basiconica [17,18]. GOBPs are expressed in sensilla basiconica from both males and females and are rather conserved among different species of moths [19]. They are therefore expected to play a key role in the recognition of generalist odorants like plant volatiles [20]. In contrast, PBPs are enriched in male antennae and have been shown to mediate transport of specific pheromone molecules [21,22,23]. However, the fact that specific odor recognition involves co-interactions between the two classes of proteins has not been excluded. GOBP2 from the silkworm moth *Bombyx mori* retains higher affinity for sex pheromones than PBPs [24]. Co-expression of various OBP homologs in the same sensilla has been shown to underlie specific olfactory coding in moths and flies [25,26,27,28].

A third class of OBP, sharing only 12–20% identity with PBPs and GOBPs, has been described in moths; these proteins of unknown function are referred to as antennal binding protein X (ABPX) [29]. ABPX-like proteins have been found in many other insect species such as flies, mosquitoes and scarab beetles, suggesting a general function or a function tuned to a very generalist odor for ABPXs [30,31,32,33,34].

Migrant insect species such as the black cutworm moth, *Agrotis ipsilon*, are likely exposed to different natural environment in which odorants from various host plants (allelochemicals) are very attractive fragrant cues [35]. This noctuid species has invaded the American continent where it has been trapped from potato corn and strawberry fields, while it feeds preferentially on corn crops in Europe, and in particular in France [36,37,38].

**Figure 1:**
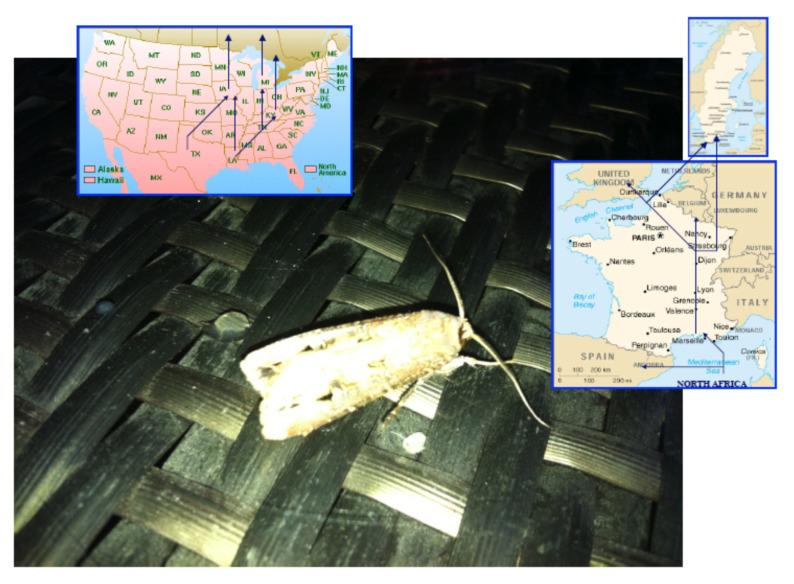
Migratory routes of *Agrotis ipsilon* on American and European continents.

Like other migrant species of moths, it migrates to different locations on both continents depending on seasons and hormonal conditions [39,40,41]. During migration, many different bouquets of plant odor molecules can be identified as potential food sources that are very attractive for the various populations of these polyphagous lepidopteran species [42]. It is well known that *A. ipsilon* is attracted during its northern migration by various spring and early summer blooming plants (including linden, *Tilia americana*; wild plum, *Prunus serotina*; crabapple, *Malus spp*; and common lilac, *Syringa vulgaris*) [42,43]. Moreover, recordings of antennal activities exposed to odors have revealed that floral volatiles from these plants, and heptanal in particular, elicited responses in both males and females [43,44]. Therefore, different intercontinental populations of a migrant moth species such as *A. ipsilon* may not be attracted by the same plant odors during their northern and/or southern flights. Pollen analysis collected from *A. ipsilon* heads revealed differential pollen source patterns between trapped spring and autumn migrant moths [45,46]. The phenomenon behind recognition and/or memorization of suitable roads or natural environments during migration remains to be determined (Figure 1).

Adult black cutworms, *A. ipsilon* (Hufnagel), from various populations are attracted by both flower odors (heptanal and phenyl acetaldehyde) and green leaf volatiles (α-humulene and β-caryophyllene) [47,48]. The pheromone blend of *A. ipsilon* has been identified in the American and French populations, both of which use the same pheromone blend, a mixture of the monounsaturated acetates (*Z*)-7-dodecenyl acetate (*Z*7-12:Ac), (*Z*)-9-tetradecenyl acetate (*Z*9-14:Ac) and (*Z*)-11-hexadecenyl acetate (*Z*11-16:Ac), but in different proportions [49,50]. This is in agreement with studies showing that Palearctic populations (France and Egypt) are different from Nearctic populations (North American) regarding pheromone production and responsiveness [51]. Gene flow between Palearctic and Nearctic populations might be minimal when compared with latitudinal gene flow. However, it could also be that the external environmental conditions strongly influence the generalist plant-odor-sensitive olfactory system of moths, without altering specific pheromone pathways.

Following the hypothesis that two intercontinental moth populations may have evolved specific olfactory elements for suitable plant odor recognition, we checked for differences between the French and American *A. ipsilon* populations with a particular attention given to OBPs. Studies on *A. ipsilon* OBPs have early focused on evolution, genomics and PBPs in the French population [52,53], but the comparison to another strain or continental population at RNA and protein levels has never been done before. In addition, the need of conducting this study was mandatory to determine which OBPs could take part in host-plant selection in a moth species that migrates thousands of miles to new crop habitats on various continents and which other gene families could be involved in the memorization of specific routes for migration and/or dispersal on suitable crops. As various populations fed on different crops, this study was also relevant to identify the basis of adaptation mechanisms on a specific parcel, which largely relies not only on plant odor recognition, but also on insecticide resistance. Accordingly, the objectives were to use a biochemical approach (electrophoresis, blotting and N-terminal sequencing) to identify population-specific OBPs among French and American populations of *A. ipsilon* and confirm the data using molecular biology. Further objectives were to bring first data on antennal protein expression and evolution of moth host plant selection, stimulate further research on how herbivorous insects accomplish the task of host plant choice and feeding ecology, better understand the mechanisms underlying orientational and feeding preferences in larvae for different plant odor sources, how the moth larvae recognize the plant on which they will grow and develop, and identify putative targets of population-specific adaptation in order to use the knowledge to develop new methods of insect pest control.

Using non-SDS PAGE and amino acid microsequencing analysis (Edman degradation), we detected two major strain-specific protein bands that were subsequently identified as *A. ipsilon* antennal binding protein X-1 (AipsABPX-1) and general odorant-binding protein-2 (AipsGOBP2), respectively. Confirming the protein data, mRNA analysis showed that ABPX was highly expressed in the French population of *A. ipsilon*, while GOBP2 was more highly expressed in the American population. No differences were found among populations in expression of two seemingly pheromone-related genes, pheromone-binding protein-1 (PBP1) and pheromone-binding protein-2 (PBP2) [34,41,49,50,51,52,53,54,55].

The novelty of this study is to compare olfactory gene expression between two strains or continental populations in a species with very known migratory routes and food/host-plant habits. This is the first molecular analysis of antennal gene expression across two different intercontinental populations for a long-life span migrant insect known to be a serious pest on many various crops worldwide. The impact is to be able to design new strategies of insect control using plant odors and targeting a specific strain of moth for which some OBP functional data are already available [52,53,54,55].

## 2. Material and Methods

### 2.1. Insect Populations

Larvae of the European strain originated from the French populations were caught in Avignon during their northward migration and subsequently reared on wheat germ until adult eclosion (INRA, Montfavet, France) according to Picimbon et al. [41,49]. Larvae of the American strain of *A. ipsilon* emerged from eggs originating from the Kansas population collected by French Agricultural Research Inc. (Lamberton, MN, USA) and reared on a corn-based black cutworm (BCW) artificial diet (BioServ, Frenchtown, NJ, USA) until adult eclosion according to Gemeno and Haynes [50]. Individuals were sexed at the pupal stage. Sexes were kept separately until preparation of tissue protein extracts. Extracts were therefore prepared from virgin *Agrotis* moths. The two populations were reared separately in these same conditions: photoperiodic 60% humidity control chambers at light:dark 16:8, 25 °C.

### 2.2. Biochemical Analysis

Adult antennae and legs were excised at their respective bases and used immediately or stored at −80 °C. Antennal and leg samples from both populations were prepared by homogenization of 40 antennae or 20 legs in 2 mM Tris-HCl/Ethylenediaminetetraacetic acid buffer (pH 7.5, 0.5 mM EDTA) and centrifugation at 12,000× *g* for 5 min at 4 °C. Proteins were then electrophoretically separated on 15% non-denaturing polyacrylamide gel (Bio-Rad, Hercules, CA, USA) and detected with Coomassie Blue or electroblotted for microsequencing following Picimbon and Leal [56]. The protein analysis was replicated in the blot. Electroblot to a glass-fiber (GF) disk was performed as described in this study [56]. Briefly, GF disks were washed in trifluoroacetic acid (TFA) for 1 h at room temperature and dried until all traces of TFA were eliminated. Immediately after the electrophoresis, the gel (15% polyacrylamide) was incubated in 1% acetic acid for 20 min and applied onto the TFA-activated GF disk between Whatman filter papers and a sponge. Blotting was performed at 350 mA (40 V) on ice for 3 h. Proteins were visualized on the GF disk by staining with Coomassie Blue. Antennal and strain-specific protein bands were cut from the GF disk and submitted for microsequencing of N-terminal amino acids (Beckman LF 3000 PS gas-phase sequenator) and sequence analysis (Swiss Protein Database of GenBank CDS).

Antennal and leg samples from both populations were also prepared using the same procedure for 15% denaturing polyacrylamide gel analysis (Bio-Rad). After electrophoresis in denaturing conditions, proteins were blotted onto a polyvinylidene difluoride PVDF membrane for N-terminal Edman sequencing following classical methods in Mini Trans-Blot electrophoretic transfer cell (Bio-Rad) [57]. Strain- and antennal-specific proteins tasks visualized by Coomassie Blue staining were selected for sequencing as described before.

### 2.3. One-Step Reverse Transcriptase Polymerase Chain Reaction (RT-PCR)

The populational specificity of OBP expression was assessed by one-step RT-PCR using total RNA as a template as described in Fujita et al. [58]. Total RNAs were extracted from antennae and legs of one-day-old adults from the French and American populations of *A. ipsilon* as previously described (TrizolTM; Life Technologies). Total RNA extracts were treated with DNAse (Fermentas) and purified by phenol extraction. Ultraviolet (UV) absorbance was used to measure RNA concentration and purity. The integrity of total RNA was further assessed by running an aliquot of the RNA sample (5 µg) on a denaturing agarose gel stained with ethidium bromide and by resolving the ribosomal RNA (rRNA). Reverse transcriptase PCR was performed using the TITANIUM one-step RT-PCR kit following the manufacturer’s recommendations (BD Biosciences Clontech). The reaction employed a combination of specific sense and antisense OBP primers:

AipsABPX-1s/5′-ATGGCGGAGCTGGCGCGC-3′,
AipsABPX-1as/5′-TATGAGGAAGTACTCGGCCTT-3′ (#AY301981);
AipsPBP1s/5′-TCGCAGGAAATCATGAA-3′,
AipsPBP1as/5′-AACTTCGGCCAAGACTTCG-3′ (#AY301985);
AipsPBP2s/5′-TCGCAGGAGGTGGTCGCC-3′,
AipsPBP2as/5′-CTATACGGCCGTCATGAT-3′ (#AY301986);
AipsGOBP2s/5′-GCATATTATAGCGCACCCC-3′,
AipsGOBP2as/5′-GTACTTCTCCATGACGGC-3′ (#AY301980) [34,52,53,54,55].

For each sample, a volume of 1 μL containing 500 ng total RNA was added as the RT-PCR template. The target transcript was reverse transcribed at 50 °C for 1 h and amplified using 40 cycles in a single reaction vessel in a common reaction buffer. The following general one-step RT-PCR protocol was used (MR Research): reverse transcription, DNA denaturation (94 °C for 5 min), three-segment amplification repeated 35 times (denaturation: 94 °C for 30 s, primer annealing: 5–62 °C for 30 s, elongation: 68 °C for 1 min) and amplicon extension (68 °C for 2 min). Finally, agarose/ethidium bromide gel electrophoresis of RT-PCR products (size of fragments: 300–600 bps) derived from interpopulational *Agrotis* tissue RNA was performed to assess the reproducible amplification of fragments encoding ABPX1, PBP1, PBP2 and GOBP2, respectively. RT-PCR products were sequenced to confirm specific identity (direct RT-PCR product sequencing).

## 3. Results

### 3.1. Interpopulational Variations of A. ipsilon Antennal Protein Profiles

**Figure 2:**
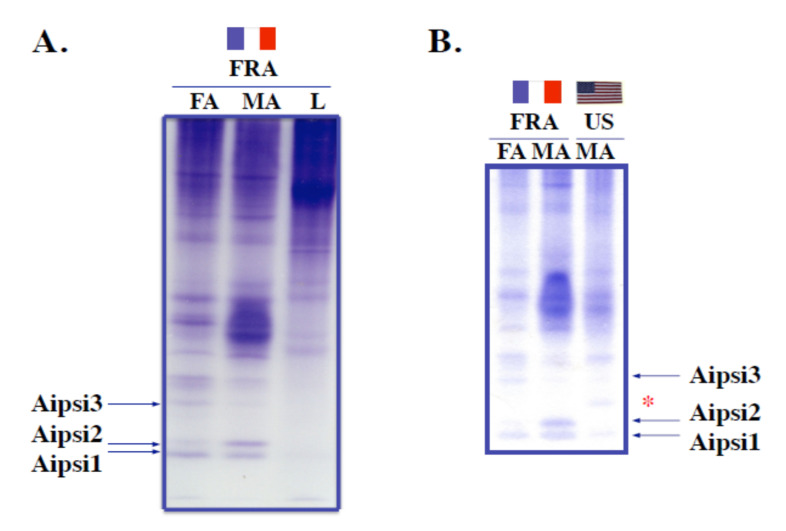
Identification of strain-specific odorant-binding proteins (OBPs) in *A. ipsilon*. (**A**) Electrophoretic comparison (15% non-SDS PAGE) of antennal soluble protein extracts (FA: female antennae, 40 eq.; MA: male antennae, 40 eq.) to leg soluble protein extracts (L, 40 eq.) from the French *A. ipsilon* population. (**B**) Electrophoretic comparison (15% non-SDS PAGE) of antennal soluble protein extracts from the French *A. ipsilon* population to antennal protein extracts from the American *A. ipsilon* population. FA: female antennae (40 eq.), MA: male antennae (40 eq.), FRA: French population, US: American population. In A and B, three protein bands, Aipsi1, Aipsi2 and Aipsi3, were stained in the zone corresponding to fast-migrating OBP-like proteins and selected for microsequencing. In B, Aipsi1 and Aipsi2 were expressed more highly in the French population of *A. ipsilon*, while Aipsi3 and another fast-migrating protein Aipsi2′ (*) were expressed more specifically in the US population.

The strain specificity of antennal soluble proteins was first assessed by analyzing the electrophoretic profiles of antennal protein mixtures from the French and American populations of *A. ipsilon* compared to leg extracts (Figure 2).

In non-denaturing (non-SDS) conditions, small soluble acidic OBP-like proteins specifically migrate to the zone corresponding to small acidic proteins at the bottom of the gel. Non-SDS-PAGE analysis of antennal extracts from the French population revealed three major protein bands (Aipsi1, Aipsi2 and Aipsi3) that were the fastest migrating proteins uniquely associated with the male and female antennal extracts (Figure 2A). Aipsi1 and Aipsi3 proteins appeared to be more abundantly expressed in females compared to male extracts, whereas Aipsi2 appeared to be more highly expressed in males (Figure 2A).

Aipsi1, Aipsi2 and Aipsi3 protein bands were blotted on GF disks and subjected to N-terminal sequencing. The N-terminal sequence obtained for Aipsi1, LTREEEANIKEAFHPFIMK, showed similarities (59–90% identity) to *A. ipsilon* OBP4 (AGR39567), *Heliothis assulta* OBP7 (AGA16511), *H. armigera* OBP7 and antennal binding protein from the tobacco budworm, *H. virescens* (AEB54591; see Table 1). The protein band corresponding to Aipsi3 gave the N-terminal motif TAEVMPHVTA specific to GOBP2s [19] (Table 1). The N-terminal sequence corresponding to Aipsi2 protein band was identified as GVVMDEDMAELARMVRESCV, showing the motif characteristic of the moth ABPXs, 9-AELA-M-R--C-19 [29,30,31,32,33,34] (Table 1 and Figure S1). The translated N-terminal amino acid sequence of AipsABPX1 (AAP578463, AY301981) matched exactly Aipsi1 (Figure S2). Analyzing Aipsi2 blot samples in both males and females showed no differences in amino acid sequences (Table 1).

Comparing male antennal extracts between American and French *A. ipsilon* populations revealed interesting differences in the profile of specific small soluble proteins (Figure 2B). In this experiment, Aipsi3 (GOBP2) was found to be more expressed in male antennal extracts from the American population compared to those from the French population (Figure 2B). In contrast, Aipsi1 was found to be strongly expressed in the French population, but to be totally absent in the American population (Figure 2B). Similarly, Aipsi2 was more expressed in antennal extracts from the French population in particular in males (Figure 2B). No trace of Aipsi2 protein was detected in the male antennal extracts from the American population. However, a protein band (*Aipsi2′) migrating in the close vicinity of Aipsi2 was rather specific to the male antennal samples from the American population (Figure 2B). The co-migration pattern of the two proteins in non-SDS conditions rather suggested that Aipsi2′ contained a homolog of Aipsi2. However, the limited amounts of protein were not suitable for N-terminal sequencing and therefore did not allow for Aipsi2′ identification, even for a replicate assay using 2–3 times the initial protein input.

### 3.2. Interpopulational Variations of A. ipsilon OBP-RNA Levels

**Figure 3:**
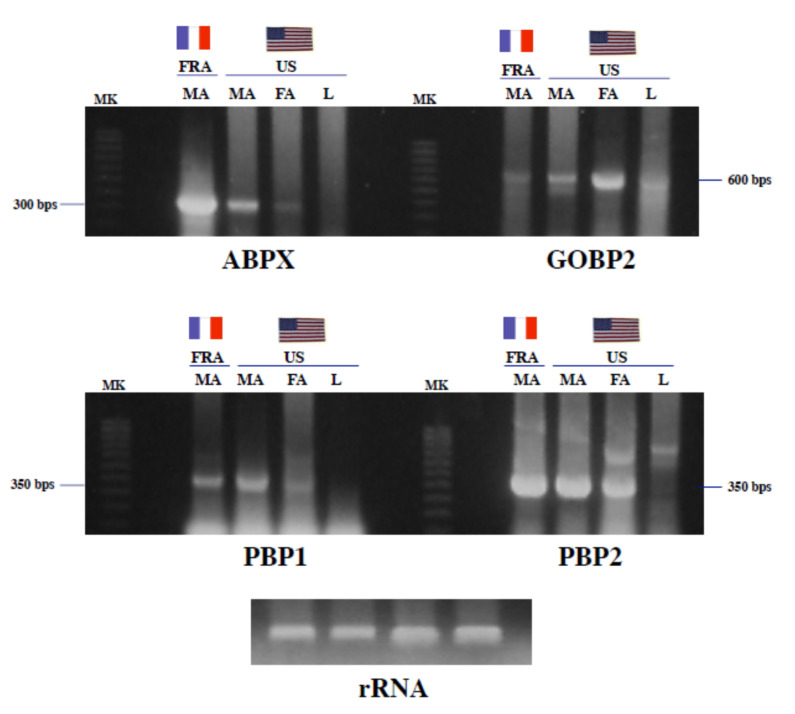
Interpopulational variations of odor binding protein-mRNA levels in *A. ipsilon*. Titanium one-step reverse transcription polymerase chain reaction analysis of total RNA (500 ng per lane) from the male antennae of the American (US) and French (FRA) populations of *A. ipsilon*. Focus was given to males since the antennal binding X (ABPX) protein was remarkably abundant in males from the French population. MK: molecular weight markers (100 bps), MA: male antennae, FA: female antennae, L: legs; total RNA from legs was used as a tissue control. For RNA control, 5 µg of each total RNA sample was analyzed on a formaldehyde agarose gel. RT-PCR products of expected size (between 300 and 600 bps) were amplified using pairwise oligonucleotide primer combinations specific to ABPX1, GOBP2, PBP1 and PBP2, respectively. PBP1 and PBP2 are used as housekeeping control genes that are expected to equally expressed across the populations. Interpopulational differences in RNA levels were noticed in ABPX and GOBP2 expression, but not in the expression of PBP1 and PBP2.

We next used the American and French continental *A. ipsilon* populations to analyze the interpopulational variations of OBP expression at the transcript level. We compared gene expression levels across different moth populations not only for ABPX, but also for GOBP2 and two pheromone-binding proteins, PBP1 and PBP2 (Figure 3).

Titanium one-step RT-PCR exhibited superior sensitivity by amplifying ABPX from the RNA samples of males from the French population (Figure 3), which is in agreement with the results obtained in the protein analysis (see Figure 2). ABPX-specific primers robustly amplified male antennal RNA from the French population. Conversely, a much smaller amount of PCR product was observed in the male and female antennal samples from the American population. No ABPX-RNA signals were detected in the leg samples (Figure 3).

Different amounts of RNA were also amplified for GOBP2 across the different population samples. GOBP2 amplification products were more pronounced in the antennal samples from the American population, particularly in females (Figure 3). No differences were found in PBP expression between the American and French populations (Figure 3). Using a specific combination of PBP1-tuned pair of primers, comparable amounts of PBP1-RNA were amplified in the antennal samples from the two populations of *A. ipsilon* (Figure 3). The same result was observed using PBP2-specific primers (Figure 3). RNA controls showed that the samples contained equal amounts of RNA (Figure 3).

### 3.3. Interpopulational Variations of A. ipsilon p38 (Calreticulin)

**Figure 4:**
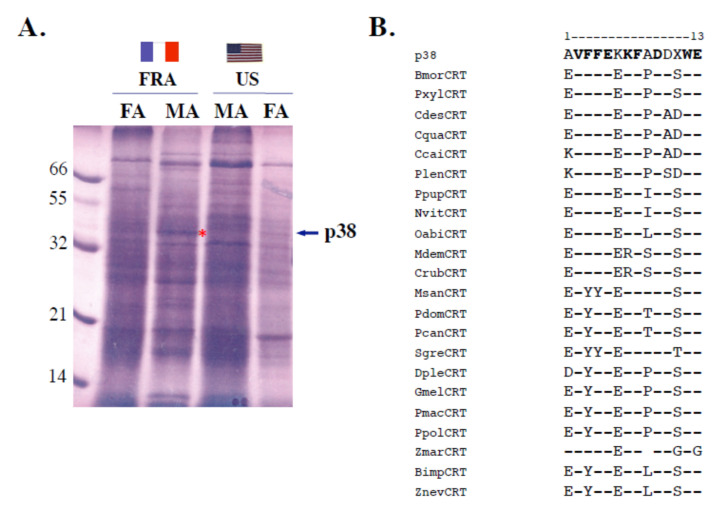
Identification of sex- and strain-specific calreticulin (p38) presence in *A. ipsilon*. (**A**) Electrophoretic comparison (15% SDS-PAGE) of antennal soluble protein extracts between the French and American *A. ipsilon* populations. MA: male antennae (40 eq.), FA: female antennae (40 eq.), FRA: French population, US: American population. The arrow indicates the position of a French population-specific soluble protein with an apparent molecular weight of 38 kDa (p38; see *). (**B**) Alignment of the N-terminal amino acid sequence of p38 (Edman degradation) with calreticulin (CRT) protein sequences from other insect species. Bmor: *B. mori* (BAC57964; NP_001037075), Bimp: *B. impatiens* (XP_003486363), Ccai: *C. cainii* (AJO70188), Cdes: *C. destructor* (AJO70005), Cqua: *C. quadricarinatus* (AIW68605), Crub: *C. rubecula* (AAN73309), Dplex: *D. plexippus* (EHJ72848), Gmal: *G. mellonella* (BAB79277), Mdem: *M. demolitor* (XP_008559929), Msan: *M. sanguinipes* (ALX00044), Nvit: *N. vitripennis* (NP_001155151), Oabi: *O. abietinus* (XP_012271064), Pcan: *P. canadensis* (XP_014616096), Pdom: *P. dominula* (XP_015184821), Plen: *P. leniusculus* (AEC50079), Pmac: *P. machaon* (XP_014371761); Ppol: *P. polytes* (NP_001298364), Ppup: *P. puparum* (ACZ68113), Pxyl: *P. xylostella* (NP_001292445), Sgre: *S. gregaria* (AEV89768), *Z. marina* (KMZ57953), *Z. nevadensis* (KDR21098). Conserved amino acids are shown in bold. X: undetermined amino acid from the p38 N terminus (Edman degradation).

By analyzing proteins via denaturing conditions, we found no apparent differences in bands corresponding to OBP proteins. In denaturing (SDS) conditions, antennal soluble OBP-like proteins migrated based on size and, therefore, could not be clearly separated. However, we found a protein with an apparent molecular weight of 38 kDa (hereafter called p38) specifically expressed in the antennae of *A. ipsilon* males from the French population (Figure 4A). Microsequencing of a PVDF blot allowed for identification of the first eleven amino acids (AVFFEKKFADD—WE). Using this short N-terminal sequence as a template to search for orthologous protein sequences in the NCBI library using blastp, we identified p38 as calreticulin. The N-terminal sequence from *A. ipsilon* p38 showed 80% identity with the calreticulin amino acid sequence from the arthropod crustacean decapods *Pacifastacus leniusculus* or *Cherax cainii*, *destructor* and *quadricarinatus* as well as from the lepidopterans *B. mori* and *Plutella xylostella* species (Figure 4B). Therefore, using two electrophoretic protein analysis methods (non-SDS and SDS conditions) coupled with blotting, Edman degradation and N-terminal sequencing identified two types of antennal proteins having similar population specificity, OBP and calreticulin.

## 4. Discussion

The American and French populations of the migrant moth species *A. ipsilon* display differences in *OBP* genes expression. Here, we found that both RNA analysis and electrophoretic comparison of antennal proteins from both populations reveal that OBPs such as ABPX are highly expressed in the French population, while some other OBPs such as GOBP2 are more highly expressed in the American population. This suggests that OBP expression could be heavily affected by external environmental conditions. There could be the possibility of the existence of some polymorphism among different strains of *A. ipsilon* in the French and/or American populations. The fact that different strains occur in *A. ipsilon* on one whole continent is not excluded. However, in our study, this is the same strain that migrates from the south of France to Sweden or from Texas/Louisiana to North America (see Figure 1). The American and French populations use the same ternary mixture for the primary long-distance pheromone recognition in the black cutworm moth [49,50,51]. Similarly, *Agrotis* hybrids only show main differences in the quantitative comparison of pheromone chemicals [59]. Different strains belonging to the same species usually show genetics and main differences in the pheromone composition as described in European corn borer moths (*Ostrinia nubilalis*, ECB: E-strain, Z-strain and E/Z or Z/E hybrids). No differences are found in pheromone-binding proteins in ECB [60].

By analyzing the profile of antennal soluble proteins in the two populations, three major protein bands corresponding to ABPX, GOBP2 and OBP4 were identified on the basis of their N-terminal sequence (see Figure 2). ABPX, GOBP2 and OBP4 are expressed in the antennae of both males and females, which suggests a general (non sex-biased) function for these proteins in agreement with previous studies [19,29,30,34,55]. The specific enrichment in ABPX of the antennae from both sexes supports the idea that ABPX mediates plant odor sensation, which constitutes specific signals that facilitate females locating oviposition and calling sites and olfactory cues for food and male recognition [17,18,19,20]. It is well known, however, that olfactory receptions of plant odors and sex pheromones are not so tightly separated; recognition of some plant odor molecules can activate specific pheromone receptor pathways [61,62]. Therefore, ABPX and some other OBPs, such as GOBP2 and OBP4, which are all highly expressed in males (see Figure 2A,B), could play a role in this phenomenon of using a pheromone system for plant recognition [63]. In *Bombyx* and *Manduca* species of moths, no labeling has been found for ABPX in male pheromone-sensitive sensilla trichodea. However, in females, ABPX has been detected in long sensilla trichodea, which are known to respond specifically to plant odor volatiles such as linalool and benzoic acid. In addition, co-expression has been found for the ABPX–GOBP2 pair [26,27]. In *Agrotis*, GOBP2 is expressed in sensilla basiconica and s. trichodea of both sexes, but no data are available for ABPX [55]. Here, there are no functional analysis results that would validate the function of the ABPX–GOBP2 pair, and *A. ipsilon* ABPX and GOBP2 proteins exhibited interpopulational differences (see Figure 2B).

Analysis of mRNA levels confirmed the results of the protein analysis between the American and French noctuid populations and showed that the two different populations of moths differ in terms of specific OBP expression such as ABPX and GOBP (see Figure 2 and Figure 3). In agreement with increased ABPX protein synthesis in the French population, ABPX mRNA levels were found to be extremely high in the French population compared to the American population in the black cutworm moth *A. ipsilon* (see Figure 3). This suggests that the expression of OBPs, and in particular ABPX, is strongly dependent on specific populational traits and/or external environmental conditions. Conversely, no differences were found between the American and French populations of *A. ipsilon* in PBP1 and PBP2 gene expression (see Figure 3), which may denote a very distinct function for *ABPX*/*GOBP* and *PBPs*.

This might be due to the fact that American and French populations of this species use a very similar sex pheromone blend [49,50,51] but need to recognize many different plant odors. The strain collected in southern France originates from North Africa and flies to Northern Europe, while the US strain trapped in Kentucky originates from Louisiana and flies northward (see Figure 1). Such a polyphagous migratory moth species certainly needs to adapt to many different environmental conditions for food selection [36,37,38]. Production and release of odors or volatile organic chemicals (VOCs) from plants are known to be heavily dependent on external factors such as biotic stresses, climate changes, night–light cycles, pollutant concentrations, seasonal variations and temperature conditions [64,65,66,67,68,69,70]. Even various genetic strains of plants have a different odor blend [71]. Plant–plant interactions are also known to be involved in specific plant odorants [72]. The odor of a plant can also be modified by the presence of fungi and other microorganisms [73,74]. However, more than light, temperature and/or the presence of bacteria and/or sister plants, the odor of a plant is strongly dependent on the presence or absence of specific herbivore species [75,76,77,78]. Therefore, it could be that two populations of moths are exposed to different plant odors due to the degree of infestation by specific herbivore organisms, and that the interpopulational variations observed in ABPX and GOBP expression levels in *A. ipsilon* reflect detection of two different blends of host-plant odor volatiles. It has been shown that last-instar moth larvae reared on a diet impregnated with citral subsequently exhibit an orientational response and feeding preferences for a citral-containing diet [79]. Similar observations have been made using diet impregnated with salt in *Drosophila* flies [80]. The phenomenon behind such innate olfactory (or taste) preferences in insects and the effects of insect larval diet on adult olfactory responses to host-plant or food volatiles are strong matters of debate [81,82]. Offspring may inherit not only their parents’ diet, but also acquire their olfactory and gustatory memory [83,84,85]. In human and mammals, it has been shown that in utero odorant exposure shapes the neuroanatomical development of the olfactory system [86,87]. Natural genetic variation also likely contributes to reprogramming of olfactory receptor neurons and odor choice, as described in the worm *Caenorhabditis elegans* [88,89]. However, the detection of differing odor sources by sibling species in moths may be reflected not only in the memorization of specific olfactory signals and new assemblies of integrative networks in the brain, but also in the expression level of the various olfactory genes involved in peripheral organs such as the antennae [90,91]. It has been shown that the acclimation to specific environmental cues requires gene transcriptional variations in olfactory receptor organs [92]. Like doves and pigeons, olfaction is crucial to homing in fishes. Odorant receptor gene expression changes during the transition from the freshwater to the marine environment in Atlantic salmons [93]. An interesting point in our study is that not only OBPs, but also calreticulin (p38) displayed antennal population specificity (see Figure 2, Figure 3 and Figure 4). In agreement with our study, an antennal soluble p38 is highly expressed in worker honeybees, *Apis mellifera* [93], suggesting not only a population- but also a caste-specific function for this protein. Calreticulin is a multi-functional protein with important roles in calcium homeostasis, molecular chaperoning and immunological response [94,95,96,97,98]. Calcium binding chaperone calreticulin has been shown to be particularly important in the behavioral response of insects, such as short-term memory in relation to integrin [99]. The high level of ABPX and calreticulin expression specifically in the French *A. ipsilon* population might therefore indicate a possible involvement of OBPs (coupled with endoplasmic reticulum resident proteins) not only in the detection, but also in the memorization of population-specific olfactory signals such as suitable host-plant odor volatiles (i.e., true indicators of migratory routes in a moth such as *A. ipsilon*).

This might be due to the fact that American and French populations of this species use a very similar sex pheromone blend [49,50,51] but need to recognize many different plant odors. The strain collected in southern France originates from North Africa and flies to Northern Europe, while the US strain trapped in Kentucky originates from Louisiana and flies northward (see Figure 1). Such a polyphagous migratory moth species certainly needs to adapt to many different environmental conditions for food selection [36,37,38]. Production and release of odors or volatile organic chemicals (VOCs) from plants are known to be heavily dependent on external factors such as biotic stresses, climate changes, night–light cycles, pollutant concentrations, seasonal variations and temperature conditions [64,65,66,67,68,69,70]. Even various genetic strains of plants have a different odor blend [71]. Plant–plant interactions are also known to be involved in specific plant odorants [72]. The odor of a plant can also be modified by the presence of fungi and other microorganisms [73,74]. However, more than light, temperature and/or the presence of bacteria and/or sister plants, the odor of a plant is strongly dependent on the presence or absence of specific herbivore species [75,76,77,78]. Therefore, it could be that two populations of moths are exposed to different plant odors due to the degree of infestation by specific herbivore organisms, and that the interpopulational variations observed in ABPX and GOBP expression levels in *A. ipsilon* reflect detection of two different blends of host-plant odor volatiles. It has been shown that last-instar moth larvae reared on a diet impregnated with citral subsequently exhibit an orientational response and feeding preferences for a citral-containing diet [79]. Similar observations have been made using diet impregnated with salt in *Drosophila* flies [80]. The phenomenon behind such innate olfactory (or taste) preferences in insects and the effects of insect larval diet on adult olfactory responses to host-plant or food volatiles are strong matters of debate [81,82]. Offspring may inherit not only their parents’ diet, but also acquire their olfactory and gustatory memory [83,84,85]. In human and mammals, it has been shown that in utero odorant exposure shapes the neuroanatomical development of the olfactory system [86,87]. Natural genetic variation also likely contributes to reprogramming of olfactory receptor neurons and odor choice, as described in the worm *Caenorhabditis elegans* [88,89]. However, the detection of differing odor sources by sibling species in moths may be reflected not only in the memorization of specific olfactory signals and new assemblies of integrative networks in the brain, but also in the expression level of the various olfactory genes involved in peripheral organs such as the antennae [90,91]. It has been shown that the acclimation to specific environmental cues requires gene transcriptional variations in olfactory receptor organs [92]. Like doves and pigeons, olfaction is crucial to homing in fishes. Odorant receptor gene expression changes during the transition from the freshwater to the marine environment in Atlantic salmons [93]. An interesting point in our study is that not only OBPs, but also calreticulin (p38) displayed antennal population specificity (see Figure 2, Figure 3 and Figure 4). In agreement with our study, an antennal soluble p38 is highly expressed in worker honeybees, *Apis mellifera* [93], suggesting not only a population- but also a caste-specific function for this protein. Calreticulin is a multi-functional protein with important roles in calcium homeostasis, molecular chaperoning and immunological response [95,96,97,98,99]. Calcium binding chaperone calreticulin has been shown to be particularly important in the behavioral response of insects, such as short-term memory in relation to integrin [100]. The high level of ABPX and calreticulin expression specifically in the French *A. ipsilon* population might therefore indicate a possible involvement of OBPs (coupled with endoplasmic reticulum resident proteins) not only in the detection, but also in the memorization of population-specific olfactory signals such as suitable host-plant odor volatiles (i.e., true indicators of migratory routes in a moth such as *A. ipsilon*).

Alternatively, it has been shown in *B. mori* that chemosensory proteins and some degradative cytochrome oxidase enzymes can be co-induced by exposure to insecticide in many various tissues, including the antennae [101]. It could be that the American and French populations of *A. ipsilon* differentially express *ABPX* and *GOBP2* genes because they have developed different insecticide resistance capacities [102,103]. A role for OBPs such as ABPX and GOBP2 in the recognition of chemical insecticides through the activation of the olfactory system (hormesis) or in the direct inactivation of insecticide molecules could have significant impacts on management. More specifically, it could facilitate the development of efficient tools to manipulate *OBP* gene expression and thereby insecticide resistance, a prelude to new powerful methods for insect pest control.

Here, we show that two different insect populations (or strains) originating from two different continents express specific *odor-binding protein* (*OBP*) genes at different levels. ABPX and GOBP2 are differentially expressed in the American and French populations of the migrant black cutworm moth, *Agrotis ipsilon*. Similar population (or strain) differences were seen with multi-functional genes such as *calreticulin*. The phenomenon behind interpopulational variations of OBP and calreticulin expression in moths remains unknown. We propose that exposure to specific plant odor volatiles and/or insecticide chemicals modulate the degree of expression in the repertoire of antennal olfactory genes. It could be a transient response or more of an epigenetic response that affects the traits of offspring. In both cases, it seems that it presents an easily testable hypothesis. Olfactory gene transcriptional variations in the insect antennae may be required for migrant species to acclimate to specific environmental conditions.

## Figures and Tables

**Table 1:** N-terminal peptide sequence identity of moth odorant-binding proteins. Conserved amino acid motifs in the N-terminus are underlined. Genus source: *Agrotis*, *Bombyx*, *Heliothis*, *Manduca*, *Sesamia* and *Spodoptera*.

Protein Band	N-Terminal Sequence	Percent Identity	Clone Name	Species Source	Access Number
Aipsi1	LTREEEANIKEAFHPFIMK	90	OBP4	ipsilon	AGR39567
		65	OBP7	*armigera*	AEB54591
		65	OBP7	*assulta*	AGA16511
		59	ABP	*virescens*	CAC33574
Aipsi2	GVVMDEDMAELARMVRESCV	100	AipsABPX	*ipsilon*	AAP57463
		89	SinfABPX	*inferens*	AGS36754
		89	HvirABPX	*virescens*	CAA05508
		89	SexiABPX	*exigua*	AGP03461
		85	BmorABPX	*mori*	CAA64446
		85	MsexABPX	*sexta*	AAF16647
Aipsi3	TAEVMPHVTA	90	AipsGOBP2	*ipsilon*	AAP57462
		90	AsegGOBP2	*segetum*	ABI24161
		90	HvirGOBP2	*virescens*	CAA65606
		90	BmorGOBP2	*mori*	CAA64445
		90	MsexGOBP2	*sexta*	AAG50015

## Supplementary Materials

The following are available online at https://www.mdpi.com/2075-4450/11/11/798/s1, Figure S1. Full-length cDNA sequence encoding *Agrotis ipsilon* ABPX (AipsABPX). (A) Nucleotide and deduced amino acid sequence of AipsABPX. The N-terminal sequence of the Aipsi2 protein band (Edman degradation) is shown in bold. The underlined regions indicate the annealing positions of specific oligonucleotide primers for one-step RT-PCR. The arrows indicate mutation sites (base changes) on ABPX clones (AAP57464, AY301982, AAP57465, AY301983, AAP57466, AY301984). Non-synonymous mutation sites are shown in red. (B) Identity between hydropathy plots (Kyte and Doolittle, window size = 15 amino acids) of AipsABPX and HvirABPX proteins. The red arrows show the position of mutation sites (in hydrophilic regions). Figure S2. Alignment of amino acid sequences from AipsABPX with other moth ABPXs and related proteins in the insect OBP family. *Agrotis ipsilon* AipsABPX (AAP57464, AY301982, AAP57465, AY301983, AAP57466, AY301984), *Sesamia inferens* SinfABPX (AGS36754), *Heliothis virescens* HvirABPX (CAA05508), *Spodoptera exigua* SexiABPX (AGP03461), *Bombyx mori* BmorABPX (CAA64446)*, Antheraea pernyi* AperABPX (CAA05509), *Manduca sexta* MsexABPX (AAF16697), *Tribolium castaneum* TcasOBP6 (EFA04594), *Rhynchophorus palmarus* RpalOBP4 (AAD31883, AAO64978), *Popillia japonica* PjapOBP1 (AAC63436), *Anomala osakana* AosaOBP1 (AAC63437), *Phyllopertha diversa* PdivOBP1 (BAA88061), *Lygus lineolaris* LAP (AAC43033), *Locust migratoria* OBP1 (4PT1-A), *Drosophila melanogaster* DmelPBP-RP3 and DmelPBP-RP6 (AAA21356, NP52424, NP524039), *Anopheles gambiae* AgamOBP1 (2ERB-A) and Agam17561 (XP321724), *Apis mellifera* ASP1 (AAD51944), *Rhynchophorus palmarus* RpalOBP2′ (AAD31883, AAO64978), *Anopheles gambiae* AgamOBP16 (AAO12082), *Zootermopsis nevadensis* OBP1 (AAN15921, KDR09910) and *Drosophila melanogaster* DmelPBP-RP1 (AAA21356, NP52424, NP524039). The six cysteine residues characteristic of the OBP family are shown in pink. The asterisks show the position of conserved cysteines. Highly conserved amino acids are indicated in blue. Residues in common between moth ABPX and counterparts in other insect species are boxed. The boxes in bold show highly conserved amino acid residues in moth ABPXs. The amino acid profile characteristic of noctuid ABPXs is shown in yellow. The N-terminal sequence of the Aipsi2 protein band (Edman degradation) is shown in red.
