# Supplementary material for: Interpopulational Variations of Odorant-Binding Protein Expression in the Black Cutworm Moth, Agrotis ipsilon
Source: Insects. 2020 Nov 13;11(11):798. doi: 10.3390/insects11110798 (PMC7696954; doi:10.3390/insects11110798)

A.

AipsABPX

ggcgtggtaatggatgaggacatggcggagctggcgcgcatggtgcgcgagagctgcgtC
G V V M D E D M A E L A R M V R E S C V

gacgagacgggcgccgacgtgaagctggtggagaaggtcaacggcggcgccgacctgatg
D E T G A D V K L V E K V N G G A D L M

gaggacgacaagctcaagtgctacatcaagtgcaccatggagacggccggcatgatgtcg
E D D K L K C Y I K C T M E T A G M M S

gacggcgaggtggacatcgaggcggtgatggcgctgctgccgcccgagatggcggagcac
D G E V D I E A V M A L L P P E M A E H

aacgggccggcgctgaaatcgtgcggcacgcagcgcggcgctgacgactgcgacacggcc
N G P A L K S C G T Q R G A D D C D T A

tggaagacgcaggtgtgctggcagaacgcgaacaaggccgagtacttcctcatatagtgc
W K T Q V C W Q N A N K A E Y F L I *

ttcacacattattataacacatcctgtccttttgattaatttttattgaacatgttttatgtc
ataatttcttactccaaattaaaattctgataaattacattttaaaaaaaaaaaaaaaaatct

B.

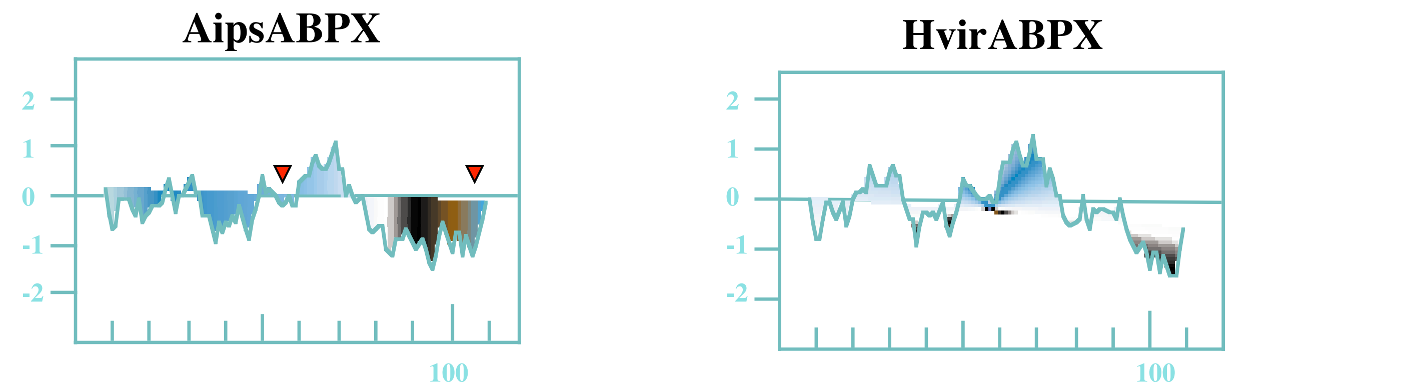

Supplement: Supplementary file 1 [file insects-11-00798-s001.zip › PicimbonInsects2020SuppFigures/PicimbonInsects2020-FigureS1.pdf]
