# Supplementary figures and images for: Interpopulational Variations of Odorant-Binding Protein Expression in the Black Cutworm Moth, Agrotis ipsilon
Source: Insects. 2020 Nov 13;11(11):798. doi: 10.3390/insects11110798 (PMC7696954; doi:10.3390/insects11110798)

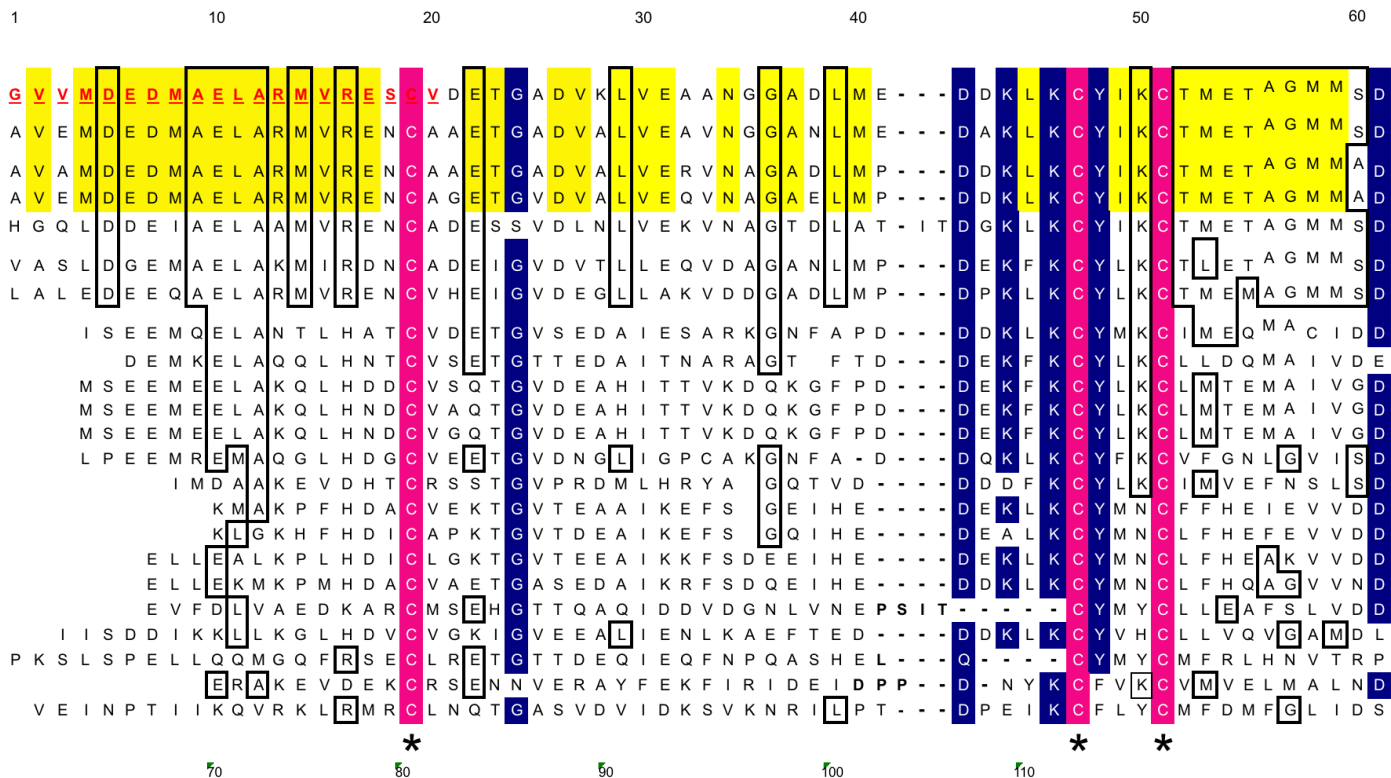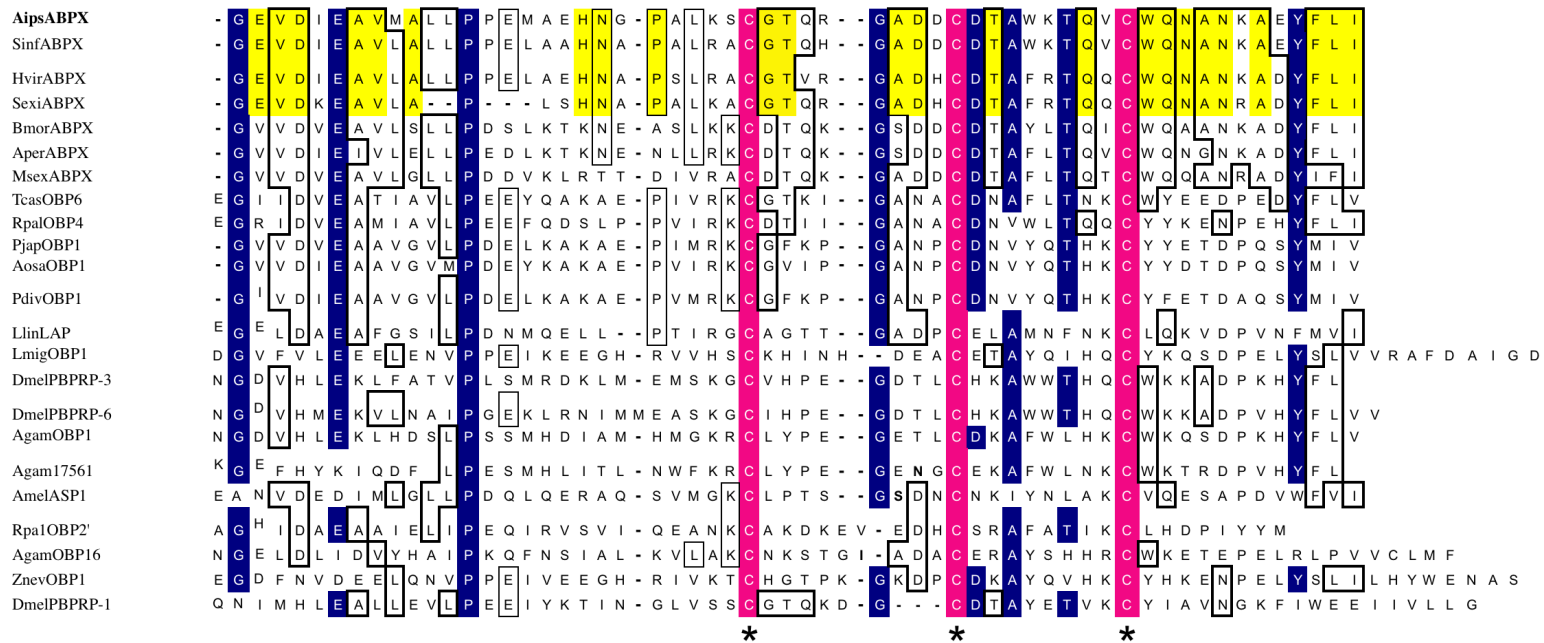

Supplement: Supplementary file 1 [file insects-11-00798-s001.zip › PicimbonInsects2020SuppFigures/PicimbonInsects2020-FigureS2 .pdf]
